# Association between Teenage Pregnancy and Family Factors: An Analysis of the Philippine National Demographic and Health Survey 2017

**DOI:** 10.3390/healthcare9121720

**Published:** 2021-12-13

**Authors:** Kozue Tabei, Erlinda Susana S. Cuisia-Cruz, Chris Smith, Xerxes Seposo

**Affiliations:** 1 School of Tropical Medicine and Global Health, Nagasaki University, Nagasaki 852-8523, Japan; cozykozue0121@gmail.com (K.T.); christopher.smith@lshtm.ac.uk (C.S.); 2 Ateneo School of Medicine and Public Health, Don Eugenio Lopez Sr. Medical Complex, Ortigas Avenue, Pasig 1604, Philippines; ecuisiacruz@ateneo.edu; 3 Department of Clinical Research, London School of Hygiene and Tropical Medicine, 15-17 Tavistock Place, London WC1H 9SH, UK

**Keywords:** teenage pregnancy, risk factors, parent structure, demographic health survey

## Abstract

Adolescence is a key developmental period in one’s life course; health-related behaviors of adolescents can be linked to lifelong consequences, which affect their future health. Previous studies highlight the role of family and its significant association with adolescents’ health. In East Asia and the Pacific, the Philippines is the only country that is showing an upward trend of teenage pregnancy while other countries in the region have declining teenage pregnancy rates. Against this backdrop, this study investigated the association between teenage pregnancy and family factors, specifically parent structure. Data for the study were extracted from the Philippine National Demographic and Health Survey 2017. All adolescent women aged 15–19 years old (*n* = 5120) were included in the analyses. The dependent variable was teenage pregnancy, while parent structure, defined as a presence or absence of parents in the domicile, was the exposure variable. Multivariable logistic regression was utilized in assessing the association of teenage pregnancy and family factors after adjusting for several potential confounders. Adolescent women were more likely to become pregnant as a teenager when they lived with neither parent (aOR = 4.57, 95% CI = 2.56–8.15), were closer to 19 years of age (aOR = 2.17, 95% CI = 1.91–2.46), had knowledge of contraception (aOR = 1.27, 95% CI = 1.22–1.32) and lived in a big family (aOR = 1.14, 95% CI = 1.09, 1.20). Furthermore, adolescent women who lived with neither parent and belonged to the poorest wealth quintile were more likely to become pregnant as a teenager (aOR = 3.55, 95% CI = 1.67–7.55). Conversely, educational attainment higher than secondary education (aOR = 0.08, 95% CI = 0.01–0.49) and those who belonged to the richest wealth quintile (aOR = 0.40, 95% CI = 0.18–0.92) exhibited a statistically inverse association with teenage pregnancy compared with those with no education and from the middle wealth quintile, respectively. Living with neither parent was found as a risk factor for teenage pregnancy. Furthermore, we found that several sociodemographic factors exhibited a non-uniform increment and reduction in the risk of teenage pregnancy.

## 1. Introduction

The United Nations Population Fund (UNFPA) estimated that 16 million adolescent women between 15 and 19 years old and 2 million under 15 years old become pregnant or give birth each year [1]. Teenage pregnancy increases the risk of maternal mortality, delivery complications, obstructed labor, systemic infections, stillbirth, premature birth, and severe neonatal complications [2,3,4]. Teenage pregnancy puts adolescents at a more significant disadvantage, including limited employment options, low educational attainment, and health drawbacks [5]. As a result, they are more likely to drop out of school than those who are not pregnant, and only a few return to school [6]. Most unmarried pregnant adolescents face immediate financial difficulty, leading to poverty. These considerable risks can cause physical impairment, sterility, mental trauma, and even death, as well as lifelong consequences such as a decrease in women’s productivity and earning capacity, which contributes to their own and their children’s poverty [7].

The global adolescent fertility rate was 42 births per 1000 women aged 15–19 in 2018. In the Philippines, the adolescent fertility rate was high at 55 births per 1000 women aged 15–19 in the same year [8]. The Philippines has the second-highest teenage pregnancy rate in East Asia and the Pacific and is the only country showing an upward trend. In contrast, other countries in the region have declining rates of teenage pregnancy [1].

Family background is one of the main risk factors of teenage pregnancy. Several studies have noted that living with both parents reduced the risk of teenage pregnancy [9,10,11]. Some concluded that teenage pregnancy was more likely to occur in adolescent women raised in a single-parent family than in a two-parent family [12,13]. Others reported that living with neither parent may lead to a high likelihood of teenage pregnancy [2,14,15].

Globally, several systematic reviews have examined the potential determinants of teenage pregnancy; however, only a few studies were conducted in the Philippines. The scarcity of evidence in the country has led to a lack of programs targeting first teenage pregnancy. Against this backdrop, this study investigated the association between teenage pregnancy and family factors, specifically parent structure. The findings can aid people working in adolescent health to understand the risk and protective factors as well as the high-risk population related to teenage pregnancy.

## 2. Materials and Methods

### 2.1. Data Preparation

Data were obtained from the Philippine National Demographic and Health Survey (DHS) 2017, a routine cross-sectional study conducted every 5 years by the United States Agency for International Development (USAID). The survey was implemented from 14 August 2017 to 27 October 2017 [16]. Here, we utilized two DHS records, household record (HR) and individual record (IR), which contain data of the Household Questionnaire and the Woman’s Questionnaire, respectively. The HR includes the basic information of typical members and visitors in the selected households. Variables within the HR records were used to select eligible women for the Woman’s Questionnaire whose ages ranged from 15 to 49 years old [17]. The IR contains seven sections, including background characteristics such as age, educational attainment, and religion. The USAID has approved use of the data following an online application on 2 April 2020.

Weighting was applied to the whole dataset to correct over- and under-sampling and restore the sample’s representativeness [18,19]. The study population was adolescent women under 20 years old who answered both the Household Questionnaire and the Woman’s Questionnaire.

**Figure 1:**
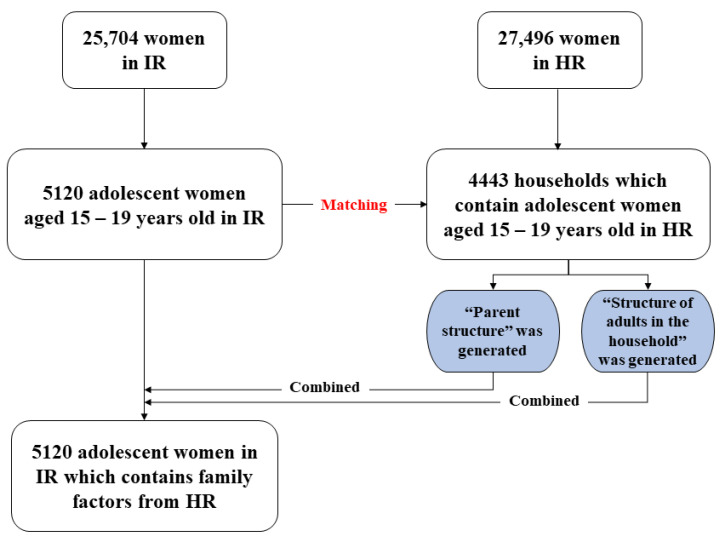
Flowchart combining data from HR to IR.

Figure 1 shows the extraction and data management flow from the HR to the IR. We restricted the age of the study population from 15 to 19 years old in concurrence with the adolescent age category and subsetted IR. Thus, the number of observations decreased from 25,704 to 5120 and only adolescent women aged 15–19 years old remained in the new IR dataset. We created a unique household ID following the DHS instruction and matched the HR and IR datasets to isolate the households with an adolescent woman. Thus, the total observations in the new HR dataset decreased from 27,496 to 4443 households. In this new HR dataset, two variables were generated and then combined with the new IR dataset, the final dataset used in our analysis.

### 2.2. Outcome Variable

The dependent variable was teenage pregnancy, either currently pregnant or having given birth regardless of the result of pregnancies by the time of the survey.

### 2.3. Exposure Variables

**Figure 2:**
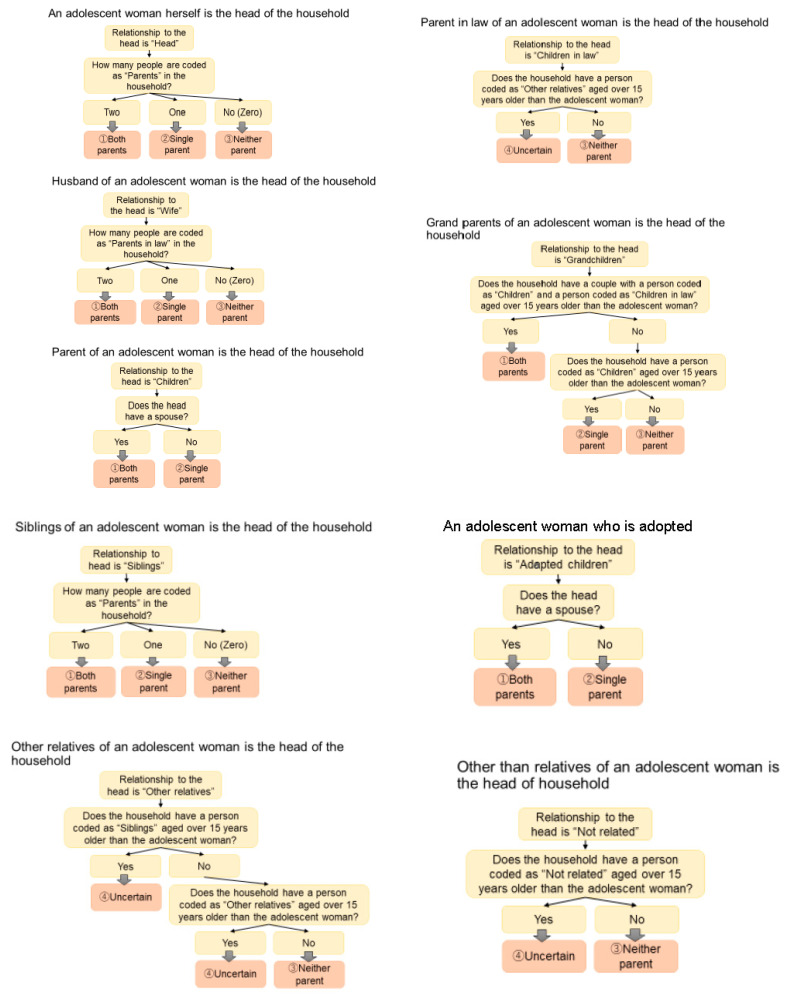
Framework in creating “Parent structure” from the HR raw data.

The exposure variable was the parent structure, representing whether a respondent lived together with both parents, a single parent, or neither parent. Since the IR dataset can only contain a woman’s individual data, it was necessary to use the HR dataset to obtain family-related data and determine whether a respondent lived together with parents. Parent structure was created based on variables in the HR raw data such as “relationship to head” (of respondents and other family members) and age of other family members (Figure 2). For example, if an adolescent woman was the head of the household, we assessed whether she lived with her parents by checking whether the person who mentioned the relationship to the head was “child.” We categorized her parent structure depending on these conditions, which are elaborately visualized in Figure 2. The same process was made for further classification according to other kinds of “relationship to the head.” When the relationship could not be identified, it was treated as a missing case.

Other potential independent variables apart from the parent structure were mainly divided into two categories: family factors and individual factors (Table 1).

### 2.4. Statistical Analysis

In univariable analysis, the distribution and frequency of each variable were shown with absolute numbers and percentages. Variables that contained more than 20% missing cases were not used for the subsequent analysis [20]. The recategorization from multilevel categorical data to binary data was also performed for several variables such as smoking tobacco and drinking alcohol. Simple logistic regression was performed to assess the association between teenage pregnancy and explanatory variables, which is commonly expressed through the odds ratio (OR) and 95% confidence interval (CI). A chi-squared test was also used to obtain a p-value, whose cutoff was set at 0.05. All tests of significance were two-sided. Variables that showed statistical significance in simple logistic regression were selected for multivariable analysis. Multivariable analysis was conducted via logistic regression modeling coupled with the forward method model simplification process. All analyses were implemented using R Statistical Programming x64 3.6.1 version [21].

## 3. Results

### 3.1. Characteristics of Teenage Pregnancy

Data from 5120 adolescent women aged 15–19 years old were obtained from the Philippine NDHS 2017. Of these, 433 women met the definition of teenage pregnancy. A total of 31.87% of the pregnant teenage respondents had both parents, whereas 11.09% and 57.04% of the pregnant teenage respondents belonged to single-parent and no-parent households, respectively. A vast majority (71.59%) of teenage pregnancies occurred in rural areas, which was nearly three times higher than the teenage pregnancies in urban areas (28.41%). In total, 42.26% of the pregnant teenage adolescents did not complete their secondary education, followed by those who completed secondary education. The proportion of pregnant teenage adolescents decreased with increasing household income.

### 3.2. Association between Teenage Pregnancy and Factors

Table 2 presents the prevalence and result of the simple logistic regression of factors associated with teenage pregnancy by sociodemographic characteristics. Teenage pregnancy was found to be 1.6 times (95% CI = 1.10–2.16) more likely to occur in adolescent women living with a single parent and 8.2 times (95% CI = 6.54–10.23) more likely in adolescent women living with neither parent compared with adolescent women living with both parents. Adolescent women in a large family showed a relatively higher tendency of having pregnant teenage adolescents (OR = 1.04, 95% CI = 1.00, 1.08). The older the age of the head of household, the less the likelihood of pregnancy among adolescent women (OR = 0.97, 95% CI = 0.96–0.97). Higher educational attainment exhibited a statistically not significant reduction in the risk of teenage pregnancy (OR = 0.26, 95% CI = 0.08–1.15). The richest and richer quintiles were less prone to teenage pregnancy, whereas adolescent women in the poorest wealth quintile were 1.8 times (95% CI = 1.34–2.34) more likely to be pregnant than those in the middle wealth quintile (richer: OR = 0.57, 95% CI = 0.39–0.83, the richest: OR = 0.37, 95% CI = 0.23–0.58). Knowledge of contraception methods showed 1.2 times greater association with teenage pregnancy than women relatively lacking such information (95% CI = 1.14–1.20).

### 3.3. Results from Multivariable Logistic Regression

The results of multivariable logistic regression of the final model with adjusted OR and 95% CIs are presented in Table 3. Teenage pregnancy was more likely to occur when adolescent women lived with neither parent (adjusted OR = 4.57, 95% CI = 2.56–8.15), who were roughly 19 years old (adjusted OR = 2.17, 95% CI = 1.91–2.46), knew about contraception (adjusted OR = 1.27, 95% OR = 1.22–1.32), and lived in a large family (adjusted OR = 1.14, 95% CI = 1.09–1.20). The risk of teenage pregnancy was reduced for adolescent women with educational attainment higher than secondary education versus no education (adjusted OR = 0.08, 95% CI = 0.01–0.49), and those belonging to the wealthiest households versus middle wealth quintile (adjusted OR = 0.40, 95% CI = 0.18–0.92). We also observed a higher likelihood of teenage pregnancy for adolescent women who lived with neither parent and belonged to the poorest wealth quintile (adjusted OR = 3.55, 95% CI = 1.67–7.55).

## 4. Discussion

### 4.1. Relation of Family Factors on Teenage Pregnancy

The current study found that teenage pregnancy was more likely to occur in adolescent women who lived with neither parent, which is consistent with published studies [2,13,15]. Two systematic reviews suggested that the absence of parents possibly increases the risk of teenage pregnancy due to decreased intra-family communication—especially regarding sexual and reproductive issues—as well as less parental monitoring, control, or guidance [22,23]. In the Philippines, a report mentioned that 27% of youth wanted to consult with their mother about sexual and reproductive issues. In contrast, less than 10% reported that sexual issues had actually been discussed in the household [24]. De Irala and colleagues [25] observed that encouraging communication between parents and children about sexual issues helps adolescents make better sexual choices. In Mpumalanga Province, South Africa, Tryphina Skosana and colleagues [26] noted that the lack of communication between parents and their children has the potential to impact sexual decision-making during adolescence. Taken together, these observations support the need to conduct further research into how the lack of a parent in the household may be linked to an unsatisfied relationship or low parental function. The current study revealed that adolescent women “who lived with neither parent” and those who belonged to the “poorest wealth quintile” were facing a significantly higher risk of teenage pregnancy than other parent structure—wealth quintile combinations (in Table 3). This suggests that the combination of wealth and parental factors may lead to even worse results. Furthermore, we found that the larger the family size, the greater the risk of teenage pregnancy, consistent with the findings from previous literature [27,28]. In Rwanda, large families (with more than 10 family members) were more than two times likely to have pregnant teenage adolescents compared with smaller households (OR = 2.13, 95% CI = 1.99–4.57) [29].

### 4.2. SES and Teenage Pregnancy

Enrollment in education higher than secondary school significantly reduced the risk of teenage pregnancy (adjusted OR = 0.08, 95%CI = 0.01–0.49), and this was consistent with other systematic reviews [6,9]. Although some studies used different criteria in categorizing educational attainment, education, in general, was linked to a lower risk of teenage pregnancy [30,31]. In a systematic review on educational attainment and teenage pregnancy in low-income countries, Mohr and colleagues [32] noted that teenage girls who had a higher education or longer educational history generally delayed pregnancy longer than teenage girls who had little or no education. Similarly, in a community-based case-control study in Ghana, Ahinkorah and colleagues [33] observed that the longer the time (i.e., in years) adolescents spend in educational institutions, the greater the likelihood of contraceptive utilization, which may be loosely related to a reduction in the risk of teenage pregnancy.

Furthermore, we also observed that adolescent women belonging to the richest wealth quintile had a reduced risk of teenage pregnancy (adjusted OR = 0.43, 95% CI = 0.19–0.97, the base is the middle quintile). Although the reference group was different, a similar study that assumed the poorest group as the reference group also noted a reduced risk of teenage pregnancy among adolescents of higher wealth status [34]. In Northern Ethiopia, Ayele and colleagues [35] observed that adolescents belonging to households with higher monthly income had a lower odds of being pregnant than those in households with low income. Although several pieces of literature have mentioned that poverty is significantly associated with teenage pregnancy [12,15,22,31,36,37], there is limited evidence on how higher wealth quintiles lead to a reduced risk of teenage pregnancy.

### 4.3. Role of Age on Teenage Pregnancy

An older age close to 19 years old was associated with an increased risk of teenage pregnancy, supported by previous studies [10,12,13]. In Uganda, the older age of participants (those aged 15–19) was also associated with the risk of teenage pregnancy, even after adjusting for several confounders [38]. In Zomba district, Malawi, Kaphagawani, and Kalipeni [39] noted that the proportion of teenage pregnancy among adolescents increased with age. The authors highlighted that the perception among teenage girls in Malawi, with the notion that they can have sexual intercourse and marry soon after the onset of menarche, is primarily influenced by cultural practices. On the other hand, in the Philippines, an adolescent who is 18 years of age is legally an adult and can exercise independence from schools and parents. This is loosely similar to that in Malawi, which highlighted the role of sociocultural factors on teenage pregnancy. It can be inferred that independence and increased freedom that comes with increasing age remotely contribute to unprotected sexual activities, leading to teenage pregnancy. However, the current study could not investigate these in-depth feelings of adolescents and thus warrants further investigation.

### 4.4. Counterintuitive Result about Knowledge of Contraception

We found a counterintuitive result whereby knowledge of contraception worked as a risk factor for teenage pregnancy. This is in contrast to the WHO systematic review, which revealed that knowledge related to contraception was a protective factor of teenage pregnancy [9,10]. We offer three possible explanations in an attempt to understand this result. First, the variable of “knowledge of contraception” was a composite variable and a sum of “yes” from 22 binary questions that had asked, “Do you know (a specific contraception)?”. Although an adolescent woman answered “yes,” it cannot be fully verified whether she knew the contraceptive method or just knew the name. This variable might not have adequately described an adolescent’s knowledge of reproductive health, which would be later connected to the succeeding possible explanation. Second, sexually active adolescents may have obtained more information regarding contraceptive methods but may have not thoroughly put them into practice. Thus, their chances of being pregnant were relatively high, regardless of their knowledge of contraception. Third, there is a possible reverse causality between teenage pregnancy and knowledge of contraception. Since they are pregnant teenagers, most of them will visit health facilities to gain knowledge about contraception. This, however, causes a reverse causal pathway whereby the outcome variable (teenage pregnancy) affects the exposure (knowledge of contraception). However, due to the limited data and instrumentation from the dataset, reverse causality was not examined.

Apart from these plausible explanations, there is a biological aspect that can provide insight into how the physiological mechanisms may be related to the gap between knowledge and practice. Brain development continues until post-adolescence through to the mid-20 s [40]. In early-mid-adolescence, the limbic system, which governs reward processing and pleasure-seeking behavior, develops before the prefrontal cortex, which controls emotions and impulsivity. This developmental disparity during early-mid-adolescence makes adolescents tend to behave and make decisions by emotions more than by rationality [3,41].

#### Limitation

The current study has several limitations. First, because of the secondary data, several potentially important data were unavailable, such as cognitive information, community and social factors, data of adolescent men, and country-specific variables such as sex education, religious activities, or social media. Though some of these variables were present in the datasets, most of these variables had more than 20% missing data. Second, due to the nature of the cross-sectional study, (reverse) causality was not examined. Moreover, since the participants were 15–19 years old at the time of when the DHS was implemented, we were not able to include those who might be pregnant later in their adolescence. Third, raw HR data were not always feasible to be categorized into parent structure, hence the existence of missing cases. Fourth, because the variable “knowledge of contraception” was a composite index, it might not reflect well and may even overestimate adolescent women’s knowledge. Amidst these limitations, the current study provides a comprehensive analysis of the role of parent structure on the risk of teenage pregnancy.

## 5. Conclusions

In the current study, adolescents who live with neither parent, particularly those living in poor households, were found to be a high-risk population for teenage pregnancy in the Philippines. Results of this study may be relevant to health managers and policy-makers alike in crafting strategies that will take into consideration how family factors, particularly parent structure, affect the risk of teenage pregnancy.

## Figures and Tables

**Table 1:** List of exposure variables.

Main Variable	Parent Structure
Family variable	
	Relationship to head Sex of household head Age of household head Number of household members Structure of adults in the household
Individual variable	
Sociodemographic variable	Age Residence Religion Ethnicity Educational attainment Wealth quintiles Marital status Current working status Occupation
Personal behavior factor	Media exposure Substance use
Family planning factors	Knowledge of contraception Knowledge of condom Current use of contraception Type of contraception currently using Contraception ever used Preferable contraception method Future intention to use contraception Perception to parental consent for obtaining contraception Access to contraception information
Sexual activities	Age of the first sexual intercourse Age of first birth Age of menarche onset Number of ideal children in the future

**Table 2:** Prevalence and the result of simple logistic regression of factors associated with teenage pregnancy by sociodemographic characteristics.

Variable	N	(%)	Teenage Pregnancy		Crude OR (CI)	*p*-Value
			N	(%)		
Parent structure						
Both parents	3364	66.09	138	31.87	Reference	
Single parent	772	15.17	48	11.09	1.55 (1.10, 2.16)	0.0111 *
Neither parent	954	18.74	247	57.04	8.17 (6.54, 10.23)	<0.001 ***
NA	30	0.59	0	0	-	-
Age					2.22 (2.02, 2.44)	0.001 ***
Residence						
Urban	1702	33.24	123	28.41	Reference	0
Rural	3418	66.76	310	71.59	1.28 (1.03, 1.60)	0.0259 *
Educational attainment (continuous)					0.85 (0.81, 0.88)	<0.001 ***
Educational attainment (categorical)						
No education	16	0.31	3	0.69	Reference	
Incomplete primary	180	3.52	55	12.7	1.91 (0.59, 8.56)	0.3286
Complete primary	186	3.63	51	11.78	1.64 (0.50, 7.35)	0.456
Incomplete secondary	3660	71.48	183	42.26	0.23 (0.07, 1.00)	0.0219 *
Complete secondary	292	5.7	97	22.4	2.16 (0.68, 9.56)	0.2391
Higher education	786	15.35	44	10.16	0.26 (0.08, 1.15)	0.0392 *
Wealth quintiles						
Poorest	1210	23.63	167	38.57	1.77 (1.34, 2.34)	<0.001 ***
Poorer	1213	23.69	112	25.87	1.12 (0.84, 1.51)	0.4482
Middle	1010	19.73	84	19.4	Reference	
Richer	894	17.46	44	10.16	0.57 (0.39, 0.83)	<0.0035 **
Richest	793	15.49	26	6	0.37 (0.23, 0.58)	<0.001 ***
Working status						
No	4187	81.78	327	75.52	Reference	
In the past year	343	6.7	51	11.78	2.06 (1.49, 2.81)	<0.001 ***
Currently working	571	11.15	52	12.01	1.18 (0.86, 1.59)	0.283
Working, but on leave last 7 days	19	0.37	3	0.69	2.21 (0.51, 6.69)	0.209
Smoking tobacco						
FALSE	5001	97.68	408	94.23	Reference	
TRUE	119	2.32	25	5.77	2.99 (1.87, 4.63)	<0.001 ***
Internet use						
Never	834	16.29	124	28.64	Reference	
Yes, last 12 month	4200	82.03	293	67.67	0.43 (0.34, 0.54)	<0.0001 ***
Yes, before last 12 months	86	1.68	16	3.7	1.31 (0.71, 2.27)	0.36
Age of menarche onset					1.11 (1.03, 1.19)	0.009 **
Knowledge of contraception					1.17 (1.14, 1.20)	<0.001 ***
Number of household members					1.04 (1.00 ^1^, 1.08)	0.0322 *
Age of household head					0.96 (0.96, 0.97)	<0.001 ***

Significance codes: 0 ‘***’ 0.001 ‘**’ 0.01 ‘*’ 0.05; ^1^ the number was rounded up.

**Table 3:** Factors associated with teenage pregnancy.

Variable	Adjusted OR	(95% CI)
Parent structure		
Both parents	Reference	
Single parent	1.49	(0.67, 3.31)
Neither parent	4.57	(2.56, 8.15)
Age	2.17	(1.91, 2.46)
Educational attainment (categorical)		
No education	Reference	
Incomplete primary	2.22	(0.38, 12.96)
Complete primary	1.88	(0.32, 10.96)
Incomplete secondary	0.31	(0.06, 1.74)
Complete secondary	0.81	(0.14, 4.62)
Higher education	0.08	(0.01, 0.49)
Wealth quintiles		
Poorest	0.93	(0.55, 1.58)
Poorer	0.89	(0.52, 1.51)
Middle	Reference	
Richer	0.71	(0.38, 1.32)
Richest	0.4	(0.18, 0.92)
Knowledge of contraception	1.27	(1.22, 1.32)
Number of household members	1.14	(1.09, 1.20)
Interaction of wealth quintiles		
Single parent * poorest	0.85	(0.26, 2.74)
Neither parent * poorest	3.55	(1.67, 7.55)
Single parent * poorer	1.01	(0.34. 3.04)
Neither parent * poorer	1.74	(0.80, 3.78)
Single parent * richer	2.63	(0.82, 8.39)
Neither parent * richer	0.59	(0.23, 1.55)
Single parent * richest	3	(0.73, 12.24)
Neither parent * richest	0.37	(0.12, 1.16)

The “*” signifies the interaction term between a specific parent structure category and a wealth quintile category.

## Data Availability

The datasets generated and/or analyzed during the current study are available upon request from the DHS program (https://dhsprogram.com/data/; accessed on 12 April 2020).
